# Determining the changes in morphology and loading status following medial displacement calcaneal osteotomy for flatfoot using patient-specific finite element models

**DOI:** 10.1038/s41598-024-65565-5

**Published:** 2024-06-26

**Authors:** Yumiko Kobayashi, Kazuya Ikoma, Masahiro Maki, Kan Imai, Masamitsu Kido, Naoki Okubo, Yasutaka Sotozono, Zhongkui Wang, Shinichi Hirai, Masaki Tanaka, Kenji Takahashi

**Affiliations:** 1 Department of Orthopaedics, Graduate School of Medical Science, Kyoto Prefectural University of Medicine, Kyoto, Japan; 2 Department of Robotics, Ritsumeikan University, Shiga, Japan; 3 Department of Anatomy and Neurobiology, Graduate School of Medical Science, Kyoto Prefectural University of Medicine, Kyoto, Japan

**Keywords:** Finite element analysis, Adult-acquired flatfoot, Patient-specific model, Medial displacement calcaneal osteotomy, Musculoskeletal system, Computational models

## Abstract

Medial displacement calcaneal osteotomy (MDCO) is the standard procedure for flatfoot. We investigated the effect of MDCO on the foot using a finite element analysis. Foot models were created from computed tomography data of 8 patients with flat feet. MDCO was performed on each model with bone translation distance of 4, 8, and 12 mm. The morphological changes, plantar pressures, and stress percentage on the talocrural and subtalar joints were evaluated before and after surgery. Morphological evaluation showed improvement in the medial longitudinal arch. The stress percentage of plantar pressure in the medial area decreased, and the stress percentage of plantar pressure in the mid- and lateral forefoot area increased. At the talocrural joint, the medial and middle stress percentage increased, while the lateral and posterior stress percentage decreased. In the subtalar joint, the stress percentage in the middle subtalar joint increased and that in the posterior subtalar joint decreased. Within the posterior subtalar joint, the anterior and medial stress percentage increased, while the posterior and lateral stress percentage decreased. Preoperative simulation using the finite element analysis may be useful in understanding postoperative morphological changes and loading conditions to perform patient-specific surgery.

## Introduction

Adult-acquired flatfoot deformity, a disease characterized by a collapsed medial longitudinal arch, forefoot abduction deformity, and hindfoot valgus that result in pain and functional depression, can be caused by various factors^1^. An appropriate treatment strategy is selected based on the mechanical impact on the foot, which can be inferred from the degree of deformity. However, the foot comprises numerous bones, ligaments, and soft tissues. Moreover, patients present with a wide variety of deformities. Thus, selecting an appropriate treatment strategy is challenging^2^.

Medial displacement calcaneal osteotomy (MDCO), an osteotomy performed to correct the deformity, remains the treatment of choice for flatfoot^3^. The medial movement and fixation of the Achilles tendon along with the calcaneus relieves the action of the triceps muscle, which contributes to external deformity, and changes the axis of rotation of the subtalar joint^4^. In a previous study, the long-term outcomes of patients who had undergone MDCO and flexor digitorum longus transfer revealed no pain or functional decline in 87% of patients and good alignment in 76%^5^.

On the other hand, it has been reported that a large amount of bone translation distance may cause lateral prominence pain at the osteotomy site and residual valgus heel alignment may cause stress on the medial ankle ligaments, but the causes of poor outcomes have not yet been investigated^6,7^.

According to international consensus, bone translation distance can range from 7 to 15 mm, based on the degree of deformity^4^. Therefore, the translation distance is determined at the discretion of the surgeon.

We hypothesized that preoperative surgical simulation would help surgeons understand the effects of MDCO, assist them in the selection of patient-specific surgical techniques and reduce the incidence of complications.

The mechanical dynamics of structures can be simulated and predicted on a computer via finite-element analysis. We used finite-element analysis to create a model based on the computed tomography (CT) data of a patient with flatfoot and perform loading simulations. The plantar pressure of the model was found to be similar to that of the patient, indicating the validity of the model^8^. MDCO performed on a flatfoot model revealed that, among the four parameters (i.e., sagittal angle, transverse angle, osteotomy position, and bone translation distance), bone translation distance had the greatest effect on plantar pressure^9^. However, only one model was used in this previous study, and the validity of other flatfoot models remains unknown. In this study, we hypothesized that the validity of other models would be good and created eight flatfoot models using the data of patients with Bluman classification stages 2A–2C to evaluate their validity and to evaluate changes in the morphology, plantar pressures, and stresses at the talocrural and subtalar joints by varying the bone translation distance during MDCO. The purpose of this study was to understand the effect of MDCO on flatfoot.

## Materials and methods

Eight patients with flatfoot deformity, comprising two men and six women with a median age of 67 (range 47 to 73) years and a median body mass index of 23.1 (range 18.8 to 26.8) kg/m^2^, who visited our hospital from 2020 to 2023 were included in this study. Patients who had previously undergone foot surgery were not included. All patients underwent CT examinations using Aquilion ONE® (Canon, Tokyo, Japan) with both hip joints at 50° flexion, both knee joints at 90° flexion, both ankle joints at neutral position, and in a non-weight bearing position (Fig. S1)^10^. The slice thickness was 0.5 mm, and the tube voltage was 120 kV. Data pertaining to the operated side were collected from the seven patients who had undergone surgery, whereas data pertaining to the more symptomatic side were collected from the remaining patient who had not undergone surgery. The severity of flatfoot was classified as follows according to Bluman’s classification^11^: stage 2A1, two feet; stage 2A2, two feet; stage 2B, three feet; and stage 2C, one foot. This study was approved by the Medical Ethics Review Committee of Kyoto Prefectural University of Medicine. (Approval no. ERB-C-1725). The recruitment period for this study is from June 5, 2020 to March 31, 2023. We confirmed that all research was performed in accordance with relevant guidelines. The patients provided written informed consent, and the data were anonymized.

### Modeling and surgical simulation

The models were created as described in a previous study^9^. The bones and soft tissue were separated from the CT data using three-dimensional reconstruction software (Ziostation 2®; Amin, Tokyo, Japan) to create standard tessellation language files. The computational load was reduced by smoothing the mesh surfaces of the bones and soft tissues and reducing the nodal points using Meshlab (http://meshlab.sourceforge.net/). The edited data were converted to standard ACIS text files and imported into Abaqus/CAE 6.14-2 (Dassault Systèmes Simulia Corp., Providence, RI, USA), a finite element software program. The imported data were rotated by the same angle as the tilt of the device and corrected such that the vertical direction from the sole of the foot was along the Z-axis.

Ligaments and plantar fascia are difficult to identify on CT images; consequently, appropriate nodes were selected as origins and insertions by referring to an anatomy textbook^12^ and created using truss elements. The cross-sectional areas were set to 18.4 mm^2^ and 58.6 mm^2^, respectively, as described by Cheung et al.^13^. External muscles, except for the Achilles tendon, were excluded as in a previous study^9^. The ground was positioned on the XY plane under the flatfoot model to simulate balanced standing. The bones and soft tissues were meshed with four-node tetrahedral elements, ligaments and plantar fascia were meshed with two-node truss elements, and the ground was meshed with eight-node hexahedral elements. Supplemental Fig. 2 provides an overview of the proposed model.

Owing to the relatively small deformations, the bones, ligaments, plantar fascia, and ground are considered linearly elastic materials. The potential force in one element, defined as U, was determined using the polynomial in Eq. (1).

1 $$U=\int \limits_{V}\frac{1}{2}{\varepsilon }^{\text{T}}D\varepsilon \text{d}V,$$

where ε is the strain vector, V is the volume, and the matrix D is the stiffness matrix depending on the elasticity (Young’s modulus) and Poisson’s ratio of the material.

Owing to the large deformation, soft tissues are considered nonlinear elastic materials of a quadratic polynomial form. The polynomial equation used for the soft tissue is shown in Eq. (2)^14^:

2 $$U=\sum_{i+j=1}^{N}{C}_{ij}{\left({\overline{I} }_{1}-3\right)}^{i}{\left({\overline{I} }_{2}-3\right)}^{j}+\sum_{i=1}^{N}\frac{1}{{D}_{i}}{\left({J}_{el}-1\right)}^{2i},$$

where I ®_1 and I ®_2 are the first and second strain invariants, respectively, C_ij and D_i are material parameters, and J_el is the elastic volume ratio. Values up to 6 can be entered for N (N = 2 in the analysis)^14^.

The values of elastic modulus (Young’s modulus) and Poisson’s ratio used for bones, ligaments, plantar fascia, and ground, and the values of material properties used for soft tissue, were similar to those in a previous study^9^.

The penalty method was used to set the contact interactions among neighboring bones and between the soft tissue and ground. Considering the lubricity of the articulating surfaces, the friction coefficient of the tangential contact between neighboring bones was assumed to be frictionless^13^. The coefficient of friction between the soft tissues and ground in the tangential direction was set as 0.6^15^. The normal contact was considered a “hard” contact, and separation after contact was allowed.

The ligaments and plantar fascia were embedded in the bone at the origin and insertion. A “tie constraint” was used to connect the soft tissues and bones. Only movement along the Z-axis was permitted for the tibia and fibula. The ground surface was immobilized completely.

In our previous study, we measured plantar pressure on one foot of a patient with flatfoot in the balanced standing and found that it was approximately half of the patient’s body weight^9^. Thus, A load of half the body weight was applied during balanced standing. Fr, the reaction force from the ground, was considered half the body weight. The tension exerted vertically upward on the Achilles tendon was considered half the value of Fr^16^. The ratio of the load exerted vertically downward on the tibia (Ft) to the load exerted vertically downward on the fibula (Ff) was set as 6:1^17^. Ft, Ff, and Ff can be expressed using Eq. (3).

3 $${F}_{t}+{F}_{f}={\frac{3}{2}F}_{r}.$$

Tension was applied to the Achilles tendon as a concentrated force in the direction of the Z-axis from a position approximating the insertion of the Achilles tendon. Loads were applied on the tibia and fibula as object forces to the entire bone in the direction opposite to the Z-axis.

MDCO was performed with bone translation distance of 4, 8, and 12 mm in all models. The standard bone translation distance was set as 8 mm in our previous study; therefore, the bone translation distance was set as 8 mm and 8 ± 4 mm in the present study^9^. The calcaneus was cut at a 45°-angle from the ground plane posterior to the insertion of the calcaneofibular ligament as described in a cadaveric foot study and the anatomy textbook^18,19^. The posterior fragment of the calcaneus was mobilized medially along the X-axis under boundary conditions. The anterior fragment of the calcaneus was fixed in all directions and immobilized. A “tie constraint” and loading simulation were used to connect the cut surfaces of the calcaneus.

### Model validation

The model was placed under loading condition. Antero-posterior view images were acquired from a point perpendicular to the XY-plane tilted 15° proximally, and the lateral view images were acquired from within the model from a point perpendicular to the YZ-plane, simulating radiographic images^20^. All measurements were acquired using ImageJ (https://imagej.net/ij/index.html).

The first to fifth intermetatarsal angle (M1M5) in the frontal plane, antero-posterior talar to the first metatarsal angle (APTM), talo-navicular coverage angle (TNC), lateral talar to the first metatarsal angle (LTM), calcaneal pitch (CP), talo-calcaneal angle (TC), and cuneiform to the fifth metatarsal height (C5MH) were measured (Fig. S3). The antero-posterior view images were measured as described in a previous study^21^. LTM and CP were measured using a method with the highest intra-observer and inter-observer correlation coefficients, even for patients with severe flatfoot^22^. The agreement between the measured foot alignment in the loaded position obtained via finite element analysis and the actual measured values obtained from radiographic images of the patient in the loaded position was evaluated.

### Evaluation of simulation results

Seven parameters used to assess the validity of the model were measured before and after surgery to evaluate morphological changes.

**Figure 1:**
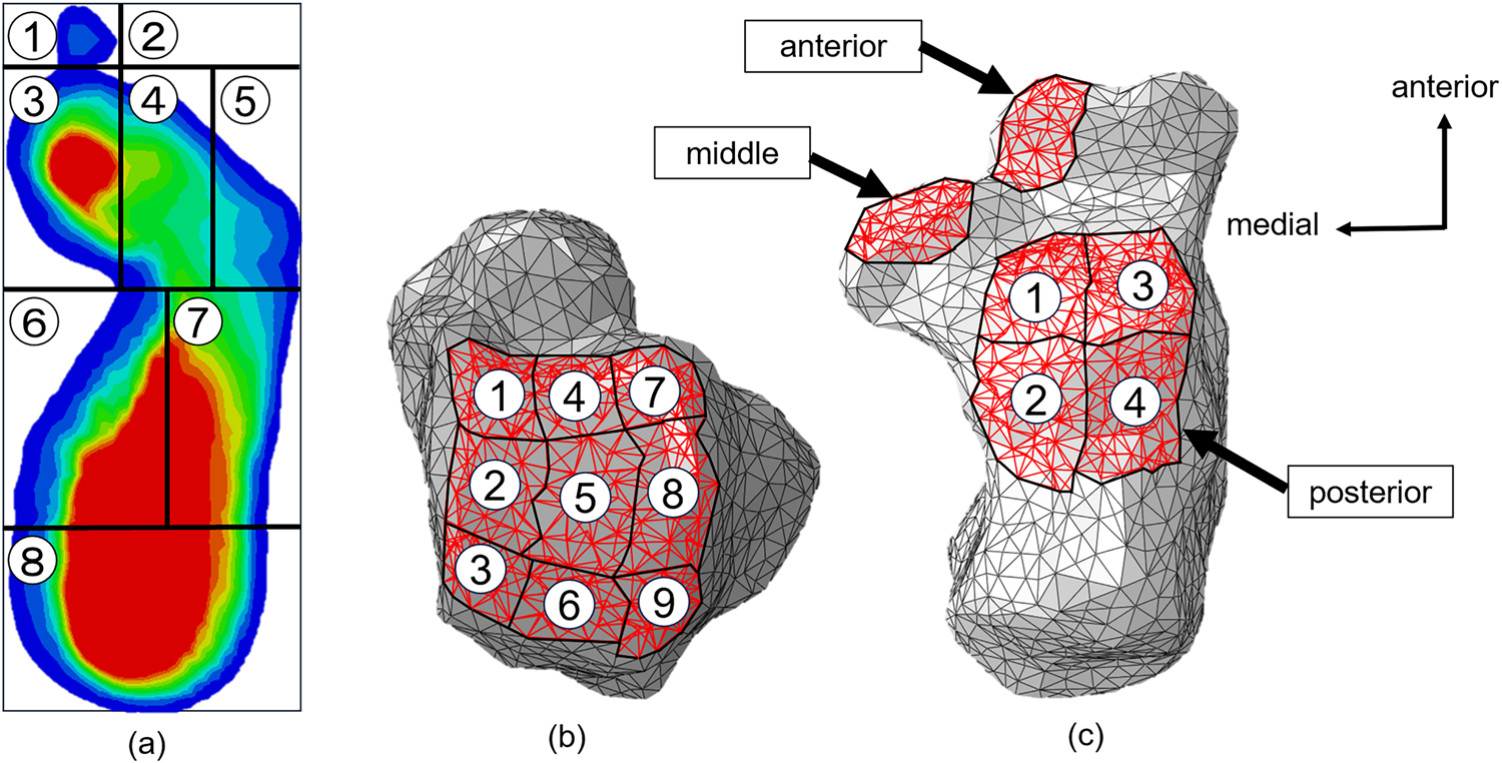
The division of the (**a**) plantar contact area (1–8), (**b**) tibiotalar joint (1–9) and (**c**) the subtalar joint (anterior, middle, and posterior are divided into 1–4).

Eight sets were created on the ground, as described in a previous study, to measure the plantar pressure^9^. Area 1 corresponded to the big toe, whereas Area 2 corresponded to the second to fifth toes. The foot, excluding the toes, was divided into three equal parts: forefoot, midfoot, and hindfoot. The forefoot was divided into Areas 3–5. The midfoot was divided into two parts, Areas 6–7. The hindfoot was designated as Area 8 (Fig. 1). The von Mises stress on each set was the output, and the percentage of the total plantar pressure was calculated to evaluate the changes pre- and postoperatively.

Sets of articular surfaces were created from the elements of the talus and calcaneus by referring to the anatomy textbook^12^. The articular surface of the talus was created using nine sets. Sets of anterior, middle, and posterior subtalar joints surfaces were created for the calcaneus. The posterior subtalar articular surface was created using four sets (Fig. 1). The number of elements in each set was equal for the joint surfaces created using multiple sets. The von Mises stresses on the elements of each set were the outputs. The percentage of the total talocrural joint in the medial (1–3), middle (4–6), and lateral (7–9) regions, as well as the anterior (1, 4, 7), middle (2, 5, 8), and posterior (3, 6, 9) regions, was calculated for the talocrural joint. The proportions of the medial (1, 2), lateral (3, 4), anterior (1, 3), and posterior (2, 4) regions to the entire posterior subtalar joint were calculated for the posterior subtalar joint. The proportions of the anterior, middle, and posterior subtalar joints, relative to the entire subtalar joint, were also calculated. The changes in the calculated proportions pre- and postoperatively were evaluated subsequently.

### Statistical analysis

All statistical analyses were performed using R software, version 3.6.3 (R Foundation for Statistical Computing, Vienna, Austria, 2018). The validity of the model was assessed using Lin’s concordance correlation coefficient (CCC) and the McBride evaluation criteria. Based on McBride evaluation criteria, the CCC > 0.99 is considered excellent, 0.95 < CCC < 0.99 good, 0.90 < CCC < 0.95 moderate, CCC < 0.9 poor.

The changes in the morphometric evaluation, plantar pressure, and stress on the articular surfaces were evaluated using the Wilcoxon signed-rank sum test. The significance level was set at < 0.0167 using the Bonferroni correction method.

## Results

The CCCs for M1M5, APTM, and TNC were 0.93, 0.95, and 0.99, respectively. All antero-posterior parameters had values of > 0.9. Among the lateral parameters, only TC had a value of < 0.9. The values of the remaining three parameters were > 0.9: LTM, 0.98; CP, 0.97; TC, 0.88; and C5MH, 0.96.

### Morphological evaluation

No significant differences were observed between the pre- and postoperative values of M1M5, APTM, TNC, LTM, CP, and TC. C5MH increased after MDCO and showed significant differences between the preoperative and MDCO4 values (p = 0.0141), preoperative and MDCO8 values (p = 0.00781), and preoperative and MDCO12 values (p = 0.00781) (Table 1).

**Table 1:** Differences between the pre- and postoperative values of the parameters.

Parameter	Preoperative	MDCO4	MDCO8	MDCO12	p-value		
	Median (interquartile range)	Median (interquartile range)	Median (interquartile range)	Median (interquartile range)	vs. 4 mm	vs. 8 mm	vs. 12 mm
M1M5 (°)	28 (23.75–31.25)	27 (23–31.25)	28.5 (23.75–30)	27 (25.5–28.25)	0.423	0.572	0.334
APTM (°)	11.5 (9.25–14.25)	11.5 (7–17.25)	9.5 (7.75–14)	9.5 (8–14.25)	0.892	0.301	0.525
TNC (°)	5 (4–17)	5 (3–16.25)	5.5 (3.75–16.5)	5 (3–17.25)	0.12	0.572	0.374
LTM (°)	− 15 (− 20.75– − 13)	− 14 (− 18.25– − 12.75)	− 13.5 (− 18.75– − 11.25)	− 14 (− 19.25– − 11.75)	0.281	0.0373	0.161
CP (°)	13.5 (9.5–15.75)	13.5 (10.75–16.75)	15 (12.25–16)	14.5 (12.25–16.75)	0.105	0.031	0.031
TC (°)	44.5 (42.75–48)	46.5 (43.5–48.25)	45 (43.75–49.5)	47.5 (44.5–49.25)	0.0975	0.289	0.0179
C5MH (mm)	4.95 (3.2–9.5)	7.85 (3.95–10.575)	9.35 (7.175–11.675)	9.7 (7.525–11.725)	0.0141*	0.00781*	0.00781*

MDCO4, MDCO8, and MDCO12, medial displacement calcaneal osteotomy with medial calcaneal translation distance of 4 mm, 8 mm, and 12 mm, respectively.

*MIM5* first to fifth intermetatarsal angle, *APTM* antero-posterior talar to the first metatarsal angle, *TNC* talo-navicular coverage angle, *LTM* lateral talar to the first metatarsal angle, *CP* calcaneal pitch, *TC* talo-calcaneal angle, *C5MH* cuneiform to the fifth metatarsal height.

*Statically significant p-value of < 0.0167.

### Evaluation of plantar pressure

Supplemental Figure 4 presents the changes in the plantar pressure in a typical case. A decrease in the medial stress and an increase in the lateral stress was observed as the bone translation distance increased. The stress percentage of plantar pressure decreased in Areas 1, 3, and 6, whereas it increased in Areas 4 and 5. Significant differences were observed between the preoperative and MDCO4, MDCO8, and MDCO12 values in these five areas (p = 0.00781 and 0.0156, respectively) (Table 2).

**Table 2:** Differences between the pre- and postoperative stress percentage of plantar pressure.

	Preoperative	MDCO4	MDCO8	MDCO12	p-value		
	Median (%) (interquartile range)	Median (%) (interquartile range)	Median (%) (interquartile range)	Median (%) (interquartile range)	vs. 4 mm	vs. 8 mm	vs.12 mm
Area 1	2.45 (1.47–3.45)	1.60 (0.88–2.64)	1.43 (0.80–1.69)	0.92 (0.29–1.28)	0.00781*	0.00781*	0.00781*
Area 2	1.28 (0.73–1.85)	1.12 (0.68–2.11)	1.32 (0.75–1.77)	0.97 (0.83–1.98)	1	1	1
Area 3	9.00 (8.24–10.65)	7.88 (6.50–9.14)	5.95 (5.02–8.53)	4.69 (3.66–7.35)	0.00781*	0.0156*	0.0156*
Area 4	9.45 (8.27–11.00)	10.99 (9.51–12.02)	12.18 (11.44–13.53)	13.43 (12.19–14.57)	0.00781*	0.00781*	0.00781*
Area 5	2.27 (1.99–2.53)	4.56 (4.19–4.93)	7.54 (4.77–8.50)	8.89 (6.70–11.47)	0.00781*	0.00781*	0.00781*
Area 6	15.93 (13.89–20.83)	14.33 (12.86–19.59)	14.13 (11.45–19.08)	12.96 (10.55–18.24)	0.00781*	0.00781*	0.00781*
Area 7	14.90 (12.49–17.11)	15.07 (11.44–16.34)	15.78 (11.80–16.87)	14.77 (11.41–16.63)	0.742	0.641	1
Area 8	41.60 (37.90–43.80)	40.31 (38.66–45.52)	39.03 (37.18–42.43)	38.27 (37.01–41.37)	0.742	0.109	0.109

MDCO4, MDCO8, and MDCO12, medial displacement calcaneal osteotomy with bone translation distance of 4 mm, 8 mm, and 12 mm, respectively.

*Statically significant p-value < 0.0167.

### Stress evaluation of joint surfaces

**Figure 2:**
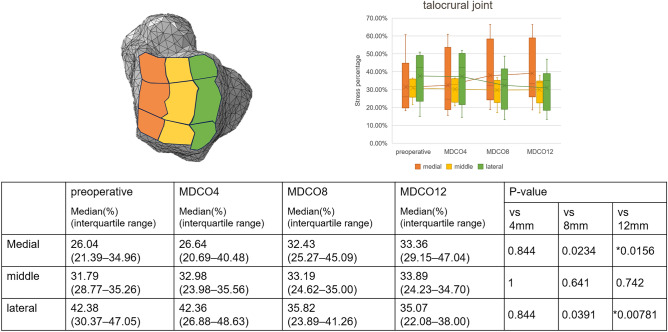
The stress percentage on the medial side increased and that on the lateral side decreased as the bone medial displacement increased in the talocrural joint. These findings indicate a significant difference between the preoperative and medial calcaneal translation of 12 mm (MDCO12) values.

**Figure 3:**
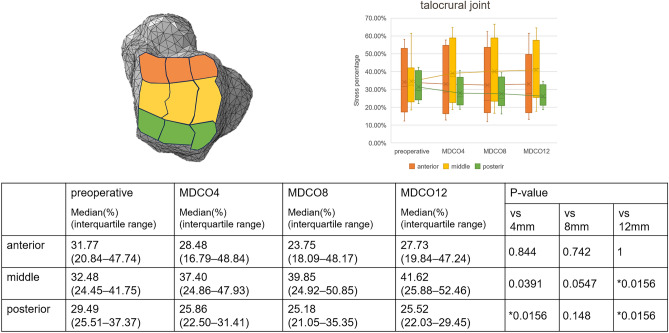
The stress percentage in the middle increased and that on the posterior side decreased as the bone medial displacement increased in the talocrural joint. These findings indicate a significant difference between the preoperative and medial calcaneal translation of 12 mm (MDCO12) values.

**Figure 4:**
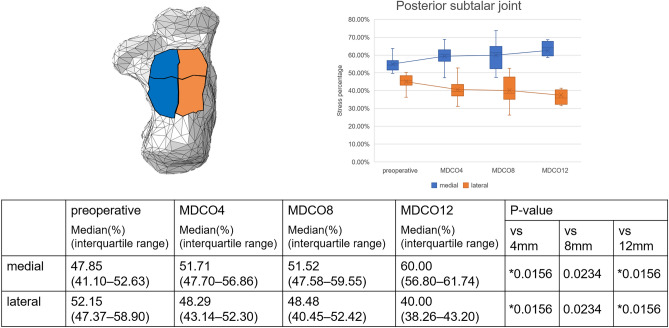
The stress percentage on the medial side increased and that on the lateral side decreased as the bone medial displacement increased in the posterior subtalar joint. These findings indicate a significant difference between the preoperative and medial calcaneal translation of 4 mm (MDCO4) values, as well as the preoperative and medial calcaneal translation of 12 mm (MDCO12) values.

**Figure 5:**
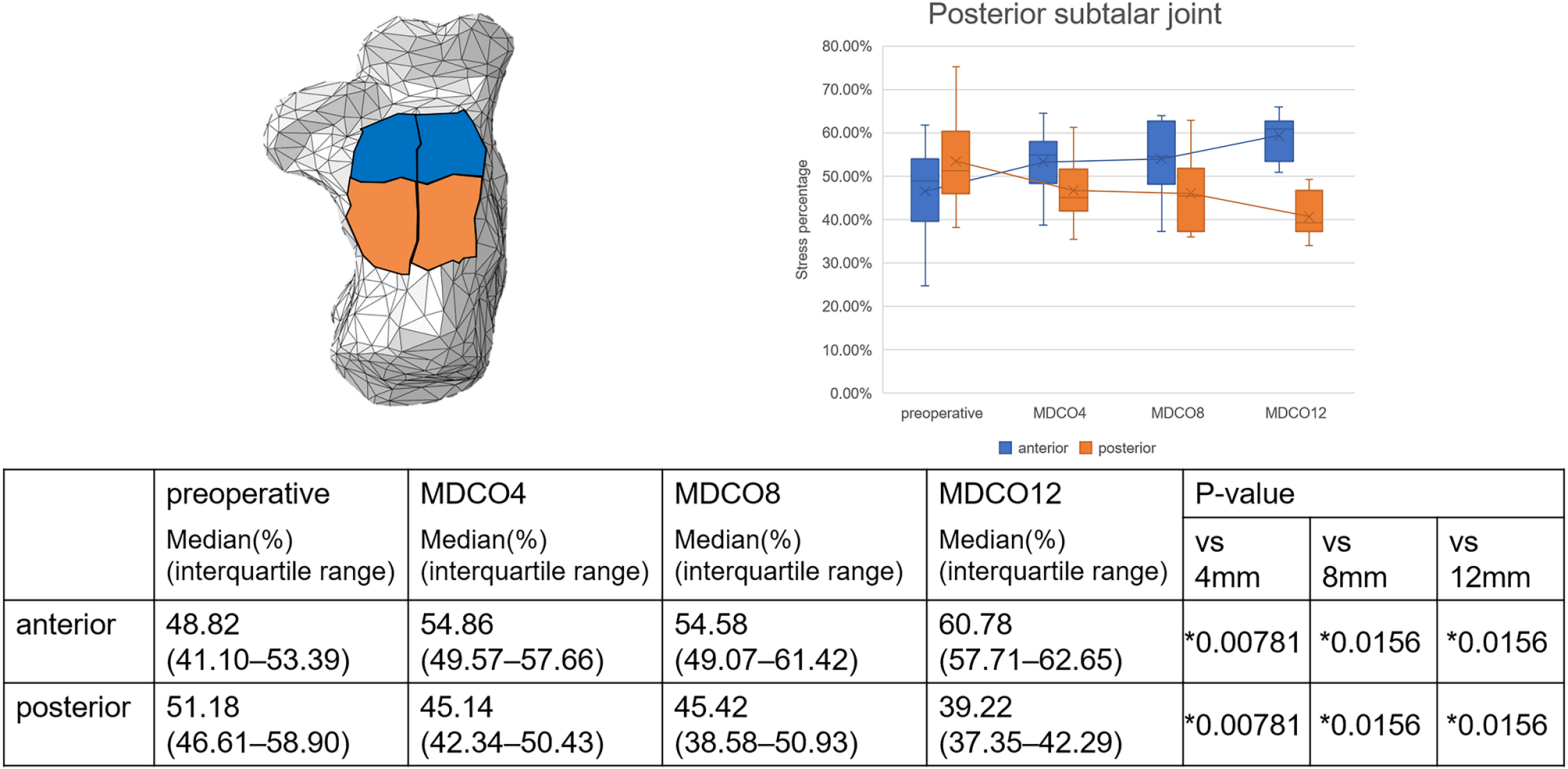
The stress percentage on the anterior side increased and that on the posterior side decreased as the bone medial displacement increased in the posterior subtalar joint increased. These findings indicate a significant difference between the preoperative and medial calcaneal translations of 4 mm (MDCO4), 8 mm (MDCO8), and 12 mm (MDCO12) values.

**Figure 6:**
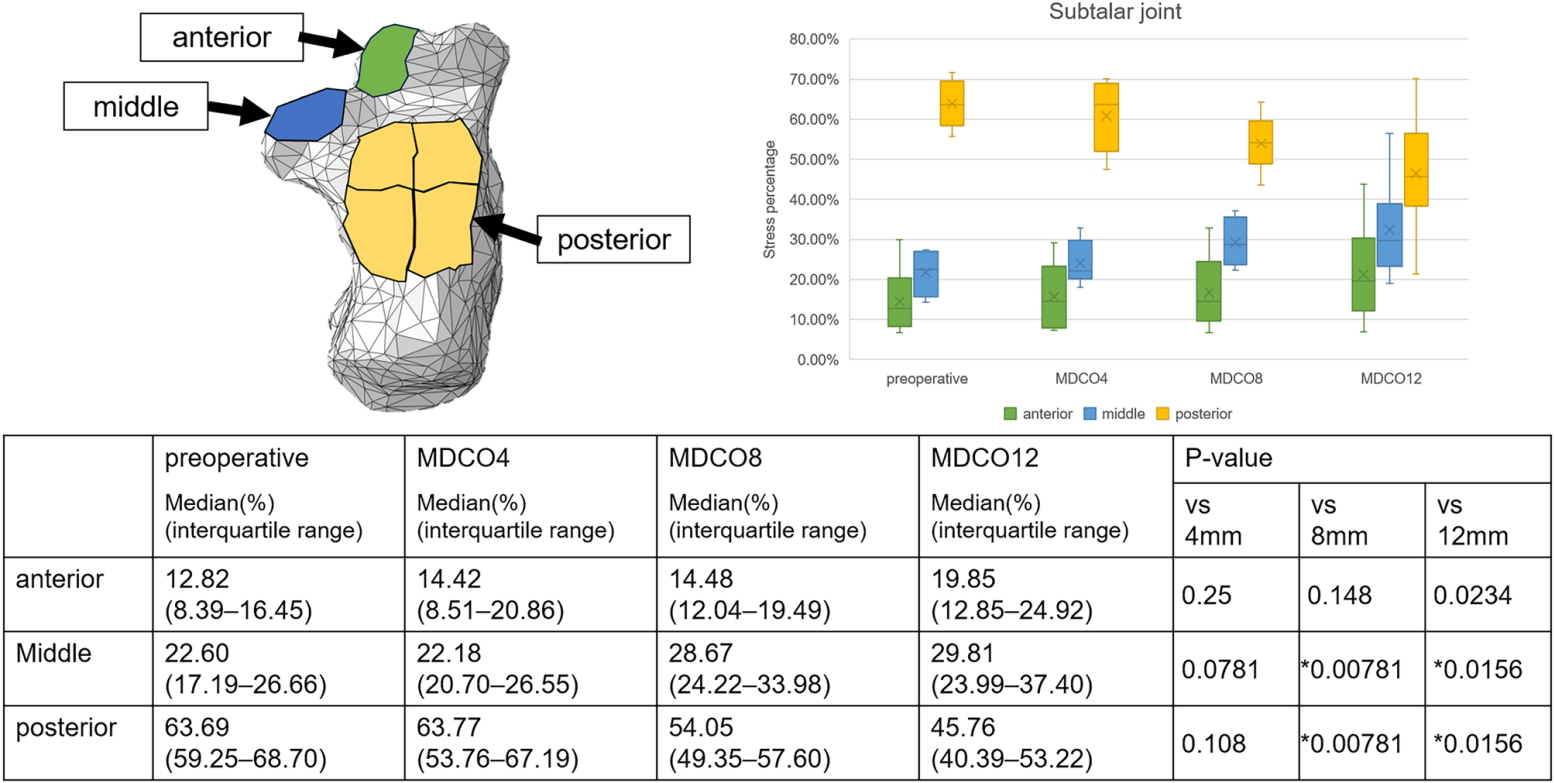
The stress percentage on the middle subtalar joint increased and that on the posterior subtalar joint decreased as the bone medial displacement increased in the subtalar joint. These findings indicate a significant difference between the preoperative and medial calcaneal translation of 8 mm (MDCO8) values, as well as the preoperative and medial calcaneal translation of 12 mm (MDCO12) values.

Supplemental Figure 5 presents the changes in stress within the joints in a representative case. The medial stress percentage increased in the talocrural joint; in contrast, the lateral stress percentage decreased. These findings indicate a significant difference between the preoperative and MDCO12 values (p = 0.0156 and 0.00781, respectively; Fig. 2). In addition, the middle stress percentage increased, and the posterior stress percentage decreased, indicating significant differences between the preoperative and MDCO4 values, as well as the preoperative and MDCO12 values (p = 0.0156; Fig. 3). The anterior and medial stress percentages increased in the posterior subtalar joint; in contrast, the posterior and lateral stress percentages decreased, indicating significant differences (p = 0.00781 and 0.0156, respectively; Figs. 4, 5). The rate of change in the antero-posterior parameters was 11.96%, whereas that in the medial and lateral parameters was 12.15%. The stress percentages of the anterior and middle subtalar joints increased, whereas that of the posterior subtalar joint decreased. The pre- and postoperative stress percentages of the middle and posterior subtalar joints showed significant differences (p = 0.00781 and 0.0156; respectively; Fig. 6).

## Discussion

Patient-specific models were created using the finite element method in this study, and MDCO was performed with three different bone translation distance to examine the morphological changes to the foot and the changes in loading conditions. To the best of our knowledge, this is the first report of a finite element study investigating the intra-articular stresses after MDCO. The morphological changes comprised an increase in C5MH. The stress percentage of plantar pressure decreased in the medial area; however, it increased in the lateral area. The medial and middle stress ratios increased in the talocrural joint; however, the lateral and posterior stress ratios decreased. Similarly, the anterior and medial stress ratios increased in the subtalar joint; however, the posterior and lateral ratios decreased. The stress percentage at the middle subtalar joint increased; however, those at the posterior subtalar joint decreased.

Previous studies have investigated the effects of MDCO on the morphology of flat foot and the changes in loading conditions^19,23–27^. A cadaveric foot specimen was created by amputating the lower leg from a healthy cadaver and separating the tendons and ligaments such that a specimen with flatfoot was attained. MDCO was performed subsequently, and the cadaveric foot was fixed to a device and loaded^19,23–27^. Radiographic images of the specimen were acquired before and after MDCO to evaluate the morphological changes^23^. The changes in the loading conditions before and after MDCO were visualized by inserting pressure-sensing sensors into the joints and under the sole of the foot^19,22–26^. However, recreating a condition similar to that of a real patient and performing multiple simulations on a cadaveric foot is difficult.

Patient-specific models that can be used to perform multiple simulations can be created via finite element analysis using patient image data. Previous studies have used finite element analysis to examine the effects of surgeries, such as MDCO and lateral column lengthening (LCL), on the foot^8,9,28^. The plantar pressures of the modeled patients and those of the model were compared in our previous study to determine the validity of the model^8^. However, measuring the plantar pressure in a stable standing position is difficult as the instability increases when the foot is pronated^29^. Thus, the use of the plantar pressure to determine the validity is inadequate. Consequently, the foot morphology of the patient was used in this study to evaluate the validity of the model. The measured values of the models after the loading simulation were compared with those of the radiographic images in the loading position. The CCC of the seven radiographic parameters ranged from 0.88 to 0.99. Furthermore, the CCC values of six of the parameters were > 0.9, indicating a high degree of agreement. Thus, the models were judged to be valid.

Various cadaveric feet and clinical studies have investigated the morphological changes following MDCO. MDCO was performed on cadaveric feet and the pre- and postoperative radiographic parameters were compared to assess the improvement in the medial longitudinal arch following MDCO in a previous study; however, no improvement was observed in the parameters assessing abduction^23^. A clinical study comparing pre- and postoperative radiographs of patients who underwent MDCO alone and those who underwent MDCO and LCL together revealed that MDCO was effective in forming the medial longitudinal arch but not in correcting abduction deformity^30^. The C5MH, which showed significant differences in this study, is the distance between the lowermost point of the medial cuneiform bone and the lowest point of the fifth metatarsal bone, an item used to evaluate the medial longitudinal arch and foot rotation^31^, and the results were similar to previous studies. Thus, the use of LCL should be considered in patients with abduction deformity.

Previous cadaveric foot studies have investigated the changes in plantar pressure following MDCO. Imhauser et al. reported that medial stress increases with foot deformity in patients with flatfoot deformity^24^. Hadfield et al. reported a decrease in the stress on the big toe and an increase in the stress on the lateral forefoot to the midfoot after MDCO in a cadaveric foot^25^. These findings are consistent with the results of the present study. In addition, Benthien et al. compared the plantar pressure achieved following LCL alone with that achieved following LCL and Cotton’s method on cadaveric feet and reported that plantar pressure in the medial forefoot region increased when Cotton’s method was used^32^. Medial column realignment, such as Cotton’s or Lapidus technique, may be required in cases where the plantar pressure in the big toe region decreases after MDCO. The use of this technique would enable the plantar side of the first metatarsophalangeal joint to be grounded. A combination of MDCO, LCL, and FDLT is generally performed in cases with stage 2 deformity^3^; however, the requirement for performing Cotton’s and Lapidus techniques additionally should also be considered. Preoperative simulation of the surgery may help determine whether realignment of the medial row is necessary.

Previous cadaveric feet studies have investigated the changes in stress within the talocrural and posterior subtalar joints. Steffensmeier et al. reported that the medial stress was increased in the talocrural joint after MDCO; however, the lateral stress was decreased, with the center of stress moving slightly anterior^26^. Davitt et al. reported that MDCO increased the medial stress and decreased the lateral stress in the talocrural joint, similar to the findings of the study by Steffensmeier et al.^19^. The posterior subtalar joint also showed an increase in the percentage of anterior and medial stresses, and the percentage change was greater than that observed in the talocrural joint^19^. These results are consistent with those of the present study. Furthermore, Michelson et al. examined the biomechanical effects of MDCO within the ankle joint using a cadaveric foot and reported internal rotation of the hindfoot and retroversion postoperatively^27^. The change in stress percentage within the joint in the present study may reflect the results of hindfoot correction.

Several clinical studies have been conducted on the middle subtalar joint. de Cesar Netto et al. reported that the percentage of patients with stage 2 flat foot who have poor coverage or open coverage of the middle subtalar joint on loading CT is about 45%, and about 5% in normal foot^33^. Lalevée et al. assessed the percentage of lateral impingement in patients with flatfoot and reported its high prevalence in the talus and calcaneus (84.9%), talus and fibula (65.2%), and calcaneus and fibula (19.4%). They also reported that a higher percentage of patients had concurrent coverage failure of the middle subtalar joint when the impingement occurred in the talus and calcaneus^34^. Thus, the proportion of stress in the posterior subtalar joint decreased, and the proportion of stress in the middle subtalar joint increased postoperatively, indicating that the loading environment of the subtalar joint was altered following MDCO. The proportion of lateral stress was high preoperatively in the posterior subtalar joint; however, it decreased after MDCO, suggesting that there was lateral impingement between the talus and calcaneus preoperatively and insufficient loading on the middle subtalar joint. The lateral impingement improved after MDCO, and the load was applied to the middle subtalar joint. The improvement in lateral impingement after MDCO suggests that the middle subtalar joint was loaded. However, an increased bone translation distance can induce high stress in the middle subtalar joint. Surgical simulation may enable researchers to study the bone translation distance in advance, thereby resulting in an appropriate intra-articular stress.

Thus, MDCO can effectively improve the morphology of the medial longitudinal arch and the loading environment of the subtalar joint. An increase in the bone translation distance may result in an excessive lateral bias of the load in some cases, which may decrease the load in the big toe region and induce high stress in the middle subtalar joint. The preoperative use of finite element analysis for surgical simulation and understanding the postoperative morphological and loading changes may facilitate the selection of patient-specific surgery.

This study has certain limitations. First, the elasticity of bone and soft tissues were uniform. The elasticity of the models used in vivo, skin, muscle, and fat vary; however, it is difficult to differentiate these structures using CT. Since the elasticity of the bone is not changed between cortical and spongy bone, there may be differences between the actual patient’s condition and the simulation results, especially in the evaluation of loading conditions^35^. In addition, Second, we added only the Achilles tendon to the models, but not other extrinsic muscles. Based on previous reports that extrinsic muscles other than the triceps and soleus complexes are not significantly affected in the balanced standing^36^, only the Achilles tendon, which transmits force to them, was added in this study. In the future, it may be necessary to consider the addition of extrinsic muscles other than the Achilles tendon when performing gait simulations. Third, we determined the insertion points of the ligaments and the plantar fascia based on an anatomy textbook. Therefore, they may differ from their original positions. Because patients with flatfoot may have damaged ligaments and it is difficult to determine the exact insertion point, and because small ligaments in the toes, even if normal, are difficult to identify on MRI, we decided to select anatomically correct positions. Fourth, the load was applied in the vertical direction to the ground. Moreover, the tibia, fibula, and Achilles tendon were loaded in this simulation and they were set to move only along the Z-axis. The restricted rotation of the tibia also restricted the alignment of the hindfoot, which may have caused the simulated results to differ from the actual foot motion of the patients^35^. However, the actual load may be applied in directions other than the Z-axis, owing to the difference between the simulation and the actual foot. Moreover, it is difficult to determine the actual degree of movement in directions other than the vertical direction and the simulation settings may become complicated. Fifth, depending on the division of the mesh of each model, the division may not be accurate when the joint surfaces are divided and evaluated. A finer mesh facilitates a more accurate division; however, it reduces the practicality of the simulation. The number of elements selected to create the set was standardized in the present study; therefore, the differences in stress measurement owing to minor differences in the area were considered to be small. Lastly, only eight patients were included in this study; consequently, proposing concrete indicators for the ideal morphological and loading conditions was not possible. Further studies with a larger sample size that use a modeling method suitable for evaluating severe cases must be conducted to develop a simulation that can recommend patient-specific surgical methods to achieve appropriate morphological and loading conditions.

In this study, we investigated the morphological, plantar pressure, and stress within the joints changes before and after MDCO using the finite element analysis. The results obtained were similar to those of previous studies, and supported our study. In the present study, simulations were performed with varying amounts of bone translation distance, and the results of plantar pressure indicated the possibility that the medial column realignment may need to be treated in addition to the MDCO when the amount of bone translation distance is large. The study of stress within the joints changes was extended to the anterior and middle subtalar joints, which provided new insight. Comparison with clinical studies suggests that MDCO may improve lateral impingement. These new findings help us to better understand the effects of MDCO.

In order to further improve this simulation method, it is necessary to verify whether this method reproduces the actual postoperative condition of the patient. In addition, by clarifying the index of the ideal loading condition, it may be possible to provide patient-specific surgical techniques tailored to the foot deformity.

## Conclusion

Models that can reproduce the morphology of patients with flatfoot were created and validated using finite element analysis in the present study. MDCO with varying bone translation distance was performed to investigate the morphological changes in the foot and the changes in plantar pressure and stress in the joint. MDCO was effective in improving the morphology of the medial longitudinal arch and the loading environment of the subtalar joint; however, it could not correct the abduction deformity. Preoperative simulation will enable the morphological changes and changes in the loading status to be determined in advance, thereby facilitating the selection of patient-specific procedures.

### Supplementary Information

Supplementary Figures.

## Data Availability

The datasets analyzed during the current study are available from the corresponding author on reasonable request.
