# Supplementary material for: Determining the changes in morphology and loading status following medial displacement calcaneal osteotomy for flatfoot using patient-specific finite element models
Source: Sci Rep. 2024 Jun 26;14:14766. doi: 10.1038/s41598-024-65565-5 (PMC11208449; doi:10.1038/s41598-024-65565-5)
Supplement: Supplementary file 1 — Supplementary Figures. [file 41598_2024_65565_MOESM1_ESM.pdf]

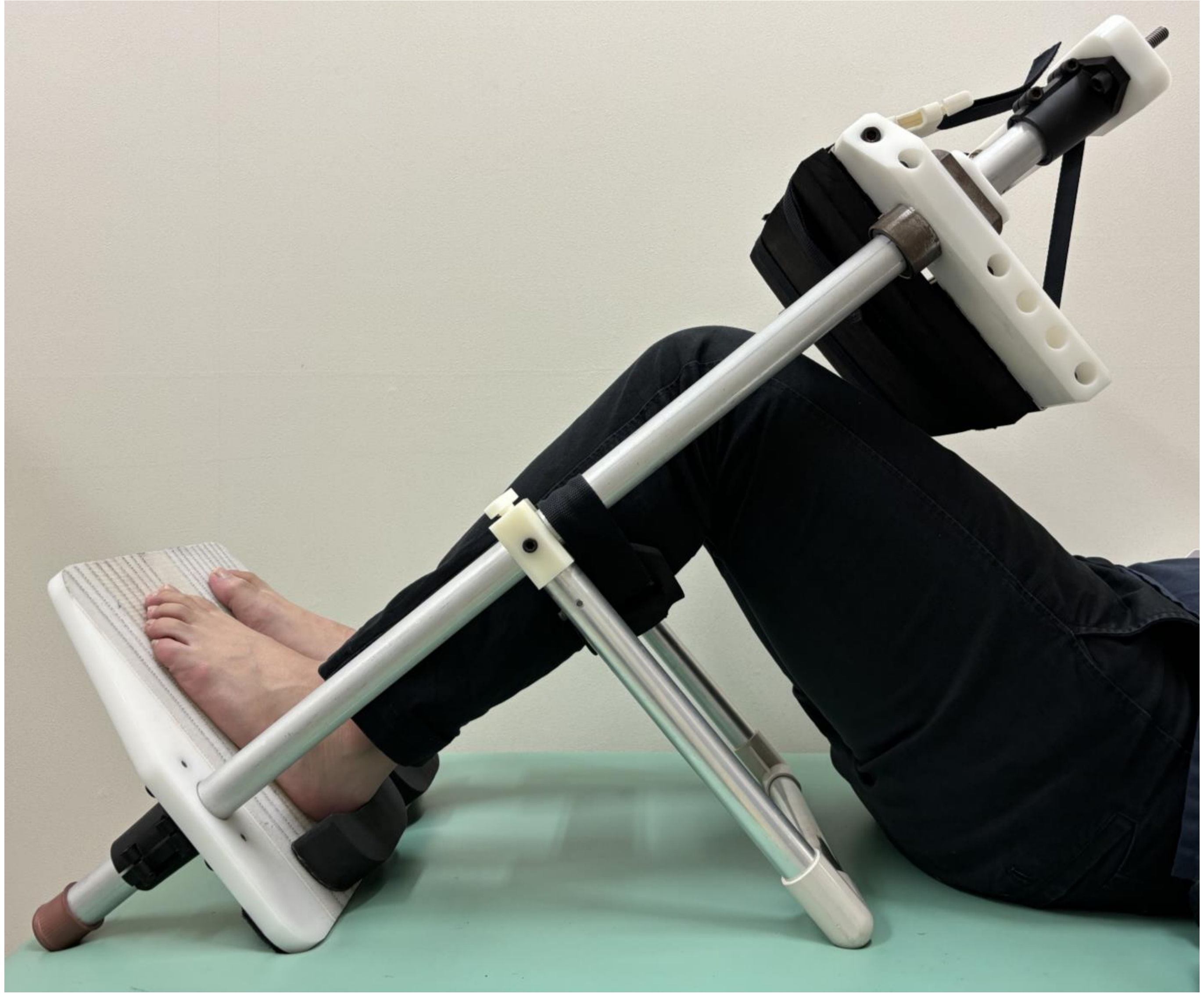

Supplemental figure. 1　Device used for computed tomography imaging

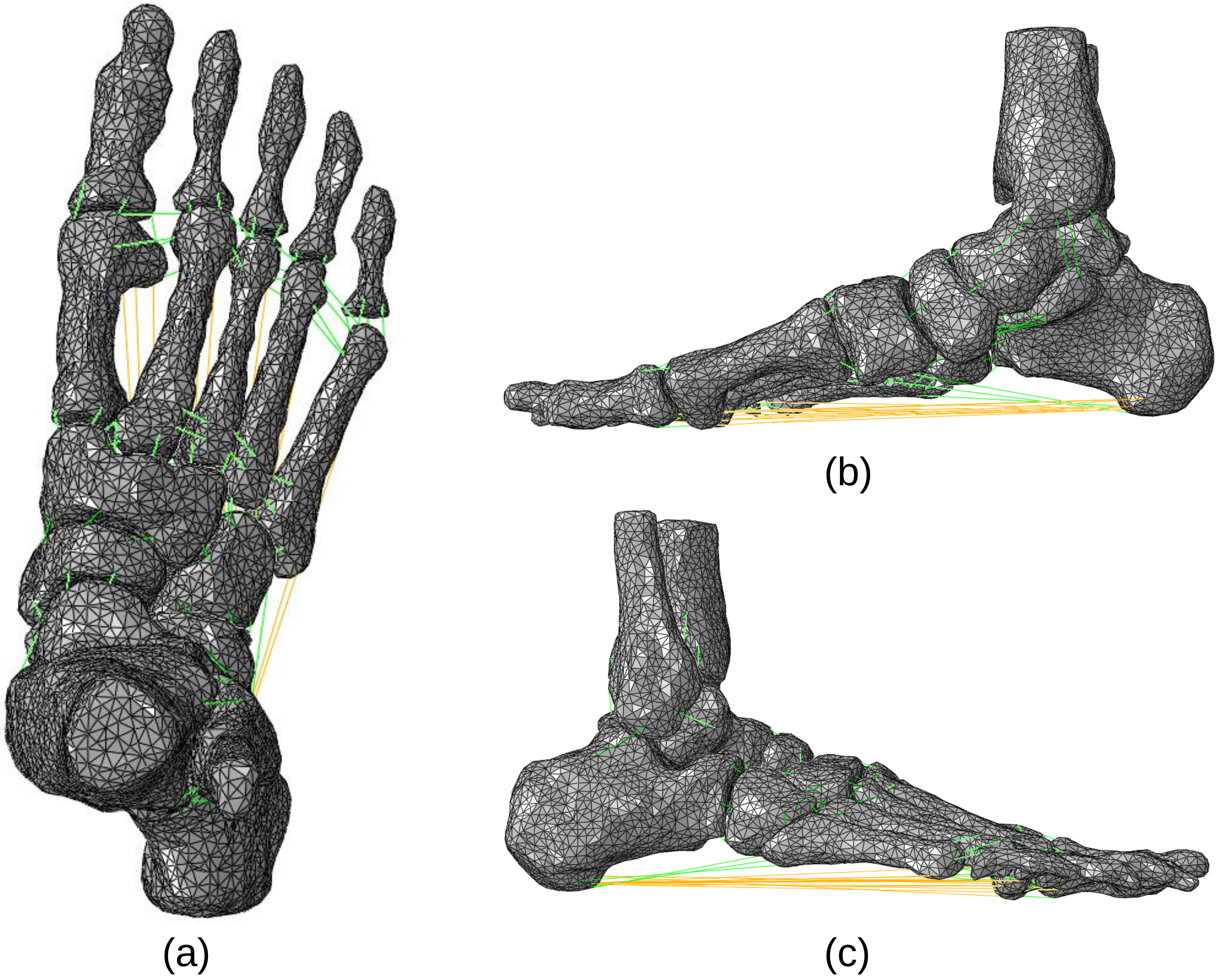

Supplemental figure. 2 Constructed finite element flatfoot model: (a) top view, (b) medial view, and (c) lateral view. The green and orange lines represent the ligament and plantar fascia, respectively.

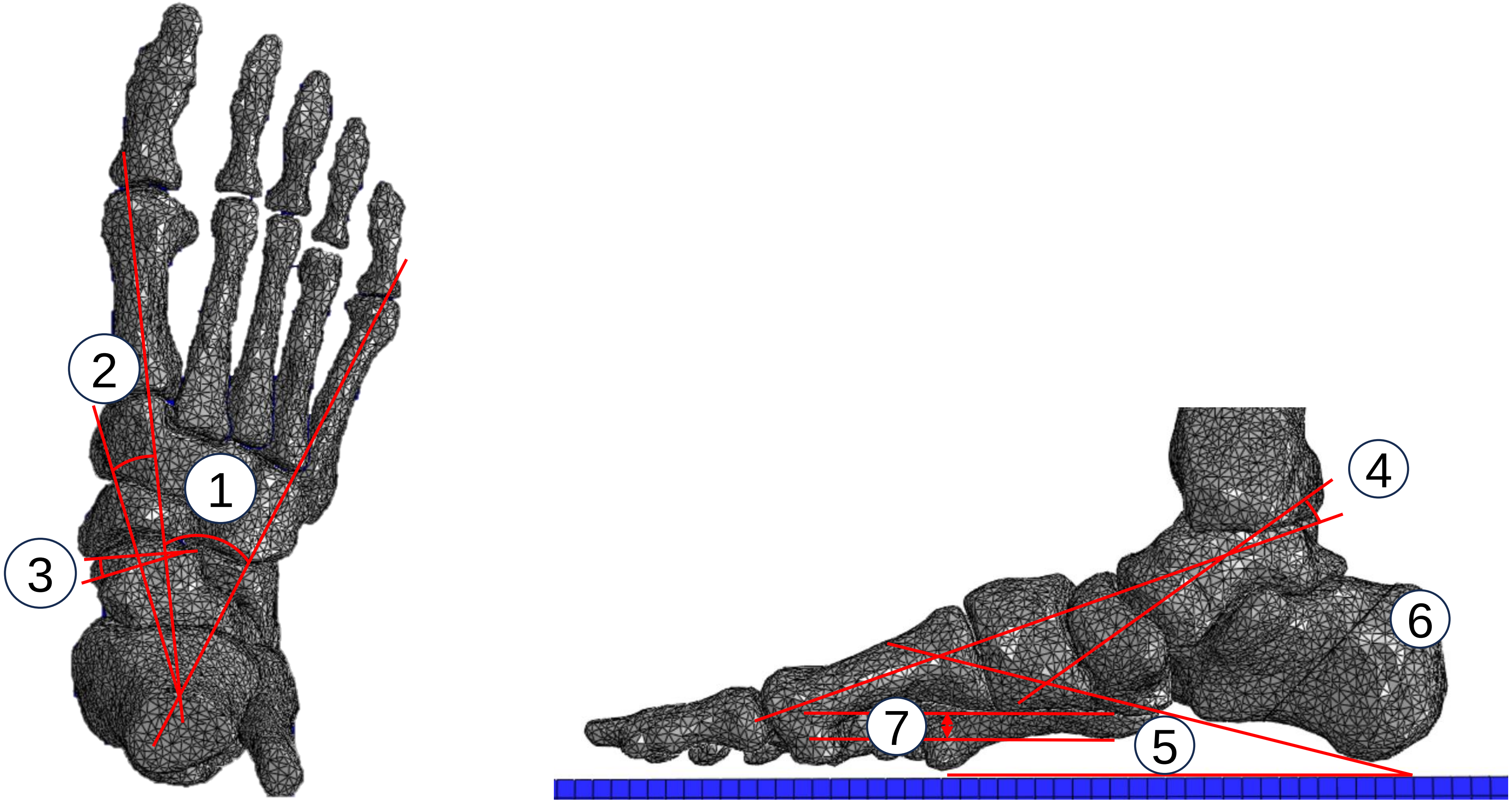

Supplemental figure 3. Measured parameters θ1: first to fifth intermetatarsal angle (M1M5), θ2: antero-posterior talar to the first metatarsal angle (APTM), θ3: talo-navicular coverage angle (TNC), θ4: lateral talar to the first metatarsal angle (LTM), θ5: calcaneal pitch angle (CP), θ6: talo-calcaneal angle (TC), Δ7: cuneiform to the fifth metatarsal height (C5MH)

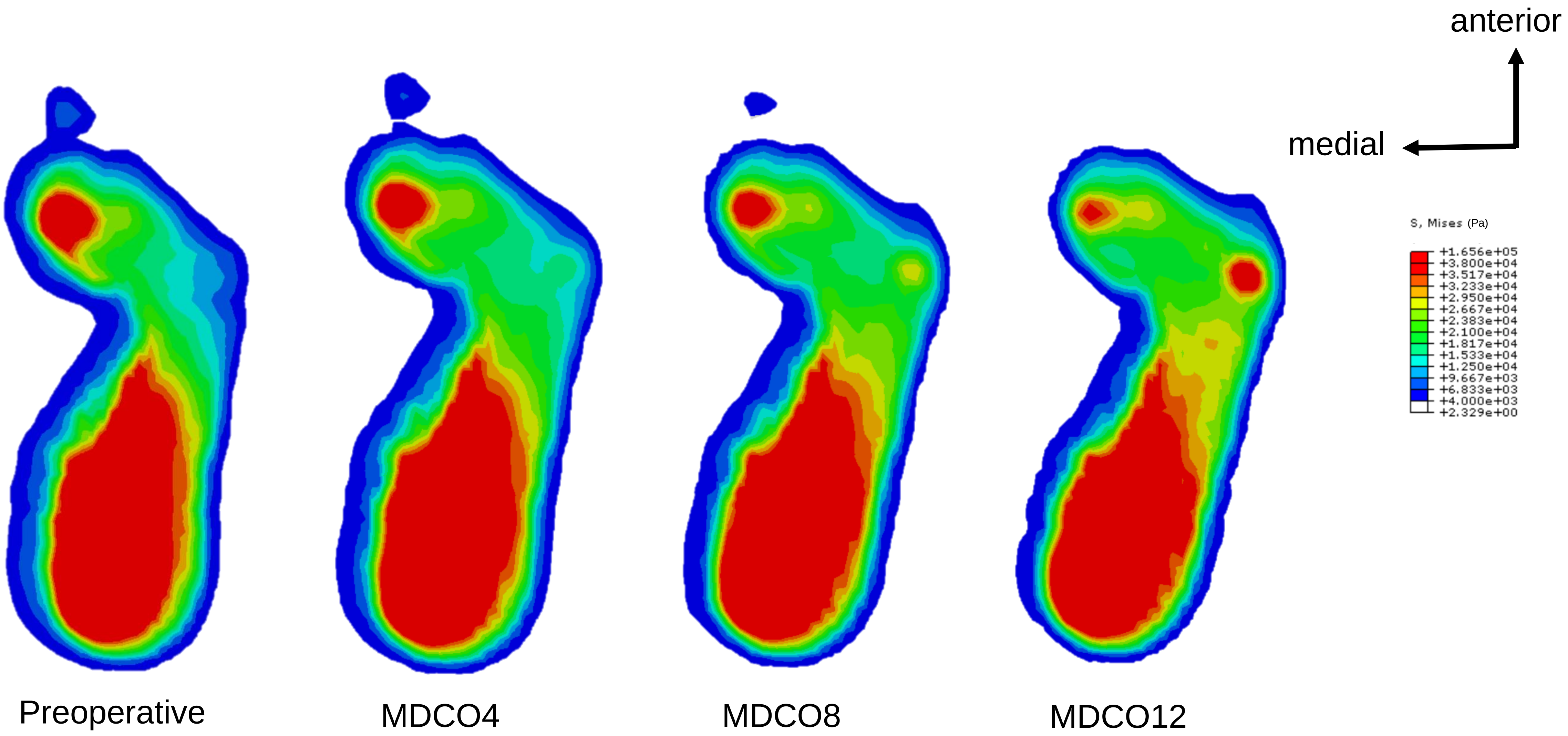

Supplemental figure. 4 A representative example of plantar pressure. The stress on the inside decreases as the medial displacement of the bone increases; in contrast, the stress on the outside increases. It is not possible to touch the ground with the big toe following MDCO12.

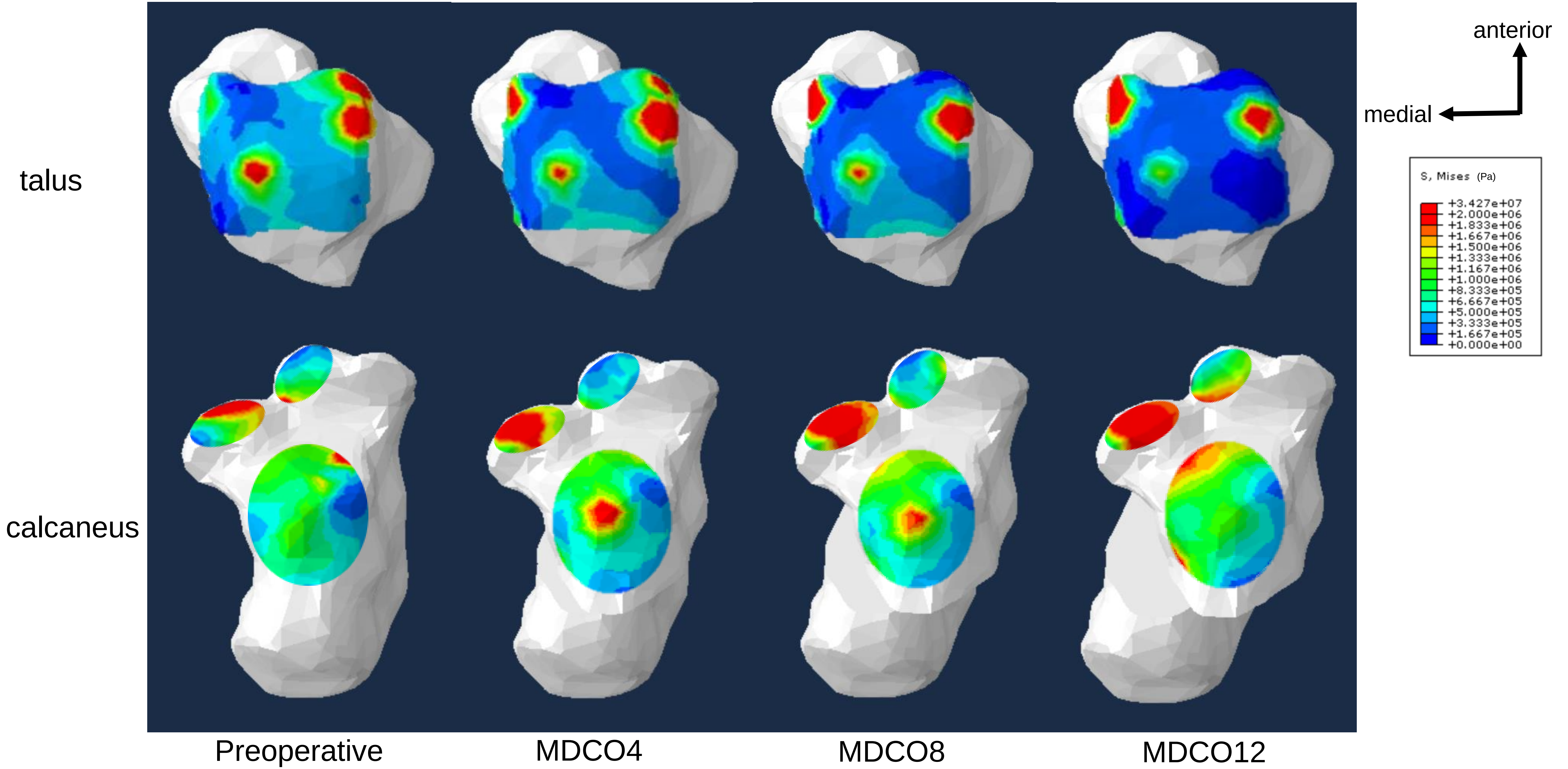

Supplemental figure. 5 Changes in stress within the joints in a representative case. The stress percentage at the center increased and that at the rear decreased. The stress percentage at the middle of the subtalar joint increased, whereas that at the posterior subtalar joint decreased. The proportion of stress in the anterior and medial areas of the posterior subtalar joint increased postoperatively, whereas that in the posterior and lateral areas decreased.
